# Zika Virus Infection Downregulates Connexin 43, Disrupts the Cardiomyocyte Gap Junctions and Induces Heart Diseases in A129 Mice

**DOI:** 10.1128/jvi.01373-22

**Published:** 2022-10-13

**Authors:** Shuxuan Li, Najealicka Armstrong, Huan Zhao, Ruth Cruz-cosme, Hongwei Yang, Chunlian Zhong, Wenkun Fu, Wei Wang, Decheng Yang, Ningshao Xia, Tong Cheng, Qiyi Tang

**Affiliations:** a School of Medicine, Henan University of Chinese Medicine, Zhengzhou, P.R. China; b State Key Laboratory of Molecular Vaccinology and Molecular Diagnostics, National Institute of Diagnostics and Vaccine Development in Infectious Diseases, School of Public Health, School of Life Sciences, Xiamen University, Xiamen, P.R. China; c Department of Microbiology, Howard University College of Medicine, Washington, DC, USA; d School of Material and Chemical Engineering, Minjiang University, Fuzhou, P.R. China; e Centre for Heart Lung Innovation - St. Paul’s Hospital, Department of Pathology and Laboratory Medicine, University of British Columbia, Vancouver, Canada; University of Kentucky

**Keywords:** Zika virus, ZIKV, A129 mouse, pathogenesis, heart diseases, flavivirus, acute myocardial injury, connexin 43, Cx43

## Abstract

Zika virus (ZIKV) is transmitted mostly via mosquito bites and no vaccine is available, so it may reemerge. We and others previously demonstrated that neonatal infection of ZIKV results in heart failure and can be fatal. Animal models implicated ZIKV involvement in viral heart diseases. It is unknown whether and how ZIKV causes heart failure in adults. Herein, we studied the effects of ZIKV infection on the heart function of adult A129 mice. First, we found that ZIKV productively infects the rat-, mouse-, or human-originated heart cell lines and caused ubiquitination-mediated degradation of and distortive effects on connexin 43 (Cx43) protein that is important for communications between cardiomyocytes. Second, ZIKV infection caused 100% death of the A129 mice with decreasing body weight, worsening health score, shrugging fur, and paralysis. The viral replication was detected in multiple organs. In searching for the viral effects on heart of the A129 mice, we found that ZIKV infection resulted in the increase of cardiac muscle enzymes, implicating a viral acute myocardial injury. ZIKV-caused heart injury was also demonstrated by electrocardiogram (ECG) showing widened and fragmented QRS waves, prolonged PR interval, and slower heart rate. The intercalated disc (ICD) between two cardiomyocytes was destroyed, as shown by the electronic microscopy, and the Cx43 distribution in the ICDs was less organized in the ZIKV-infected mice compared to that in the phosphate-buffered saline (PBS)-treated mice. Consistently, ZIKV productively infected the heart of A129 mice and decreased Cx43 protein. Therefore, we demonstrated that ZIKV infection caused heart failure, which might lead to fatal sequelae in ZIKV-infected A129 mice.

**IMPORTANCE** Zika virus (ZIKV) is a teratogen causing devastating sequelae to the newborns who suffer a congenital ZIKV infection while it brings about only mild symptoms to the health-competent older children or adults. Mouse models have played an important role in mechanistic and pathogenic studies of ZIKV. In this study, we employed 3 to 4 week-old A129 mice for ZIKV infection. RT-qPCR assays discovered that ZIKV replicated in multiple organs, including the heart. As a result of ZIKV infection, the A129 mice experienced weight loss, health score worsening, paralysis, and deaths. We revealed that the ZIKV infection caused abnormal electrocardiogram presentations, increased cardiac muscle enzymes, downregulated Cx43, and destroyed the gap junction and the intercalated disc between the cardiomyocytes, implicating that ZIKV may cause an acute myocardial injury in A129 mice. Therefore, our data imply that ZIKV infection may jeopardize the immunocompromised population with a severe clinical consequence, such as heart defect.

## INTRODUCTION

Zika virus (ZIKV), along with yellow fever (YF), Japanese encephalitis (JE), West Nile (WN), and the dengue (DEN) viruses, is characterized as a positive-sense single-stranded RNA virus and belongs to the family of *Flaviviridae* and the genus flavivirus (1–3). A growing number of strains of ZIKV have been isolated from more than 60 countries (4, 5). ZIKV is a zoonotic pathogen that circulates among the animals and humans via mosquitos in the tropical or subtropical areas. Although it caused epidemic outbreaks in Micronesia, French Polynesia, Cook Island, and Easter Island (6), it was the 2015 to 2016 pandemic of ZIKV infection in South America, especially in Brazil, that caught the public attention. The transmission and the epidemic of ZIKV have been now under control, but its infection may reemerge due to it being mosquito borne and the lack of an effective vaccine. Therefore, studying and understanding its pathogenesis are still necessary.

ZIKV infection has been related to the increasing number of cases of microcephaly and Guillain-Barré syndrome (GBS) in the areas of the epidemics (4, 7, 8). Currently available pathogenic insights have been largely obtained from two different mouse models: immunocompromised adult mice and neonatal mice. ZIKV-infected pregnant women can transmit the virus to their feta and cause neuronal damage, especially in the first trimester, suggesting that ZIKV is able to cross the placental barrier of humans. However, the placental barrier of a healthy mouse has a different structure from that of human so that the transmission of ZIKV to fetus in normal mouse could not be detected (9). Plus, the normal mice have failed to be productively infected by ZIKV, although the wild type (WT) adult mouse is important for ZIKV study (10–12). The mice used as models for ZIKV studies so far have deficiencies in either the interferon (IFN) or the IFN receptor which are caused either genetically or via antibody treatment (13). The A129 mouse has genetically knocked out its receptors for type I IFN (IFN alpha and beta) and hence is not susceptible to stimulation of IFN alpha and beta, thus it is vulnerable to ZIKV infection (14). The other mouse models include the AG129 mouse that lacks the IFN-alpha, -beta, and -gamma receptors (14, 15), and the triple knockout mouse with knockout of three genes (*irf3^−/−^*, *irf5^−/−^*, and *irf7^−/−^*) (15).

Thus far, the WT mice that were used for ZIKV infection were mostly, if not all, neonatal ones (16–18). One-day-old to 3-day-old WT C57BL/6 mice were shown to be infected with ZIKV and developed symptoms (17). We recently systemically investigated the ZIKV infection in neonatal mice and presented the outcomes in pathogenesis, virus-host interaction, and development of neuronal damages (18). The results of ours and other groups showed that ZIKV not only replicates in WT neonatal mice, but also causes deaths of these mice (15–21). ZIKV infection in 1-day-old to 3-day-old mice causes 100% fatalities (18). It has remained unknown whether ZIKV induces heart failure in older mice. Although the immunocompetent WT mice are resistant to ZIKV infection, ZIKV infection in adult AG129 mice also causes a high mortality (19, 22–24). Therefore, we wonder whether ZIKV causes heart diseases in 3- to 4-week-old A129 mice. To address this issue, in this study we employed A129 mouse models to investigate the viral pathogenesis. We found that ZIKV replicated in multiple organs of 3- to 4-week-old A129 mice after infection, induced significantly higher levels of creatine-kinase (CK), CK-MB, lactate dehydrogenase (LDH), alpha-hydroxybutyrate dehydrogenase (α-HBDH), cardiac troponin T (cTnT), and cardiac troponin I (cTnI) and abnormal electrocardiogram (ECG) presentations that are the biomarkers for acute myocardial injury, decreased the gap junction protein Cx43, and altered the structure of the intercalated disc (ICD). ZIKV caused fatal infection of the A129 mice, which is consistent to the previous report. We also revealed that ZIKV infection induced heart defects, which may lead to the deaths of the mice.

## RESULTS

### ZIKV productively infects human, murine, and rat cardiac muscle cells and Cx43 protein is decreased by ZIKV infection in the cardiomyocyte.

**FIG 1:**
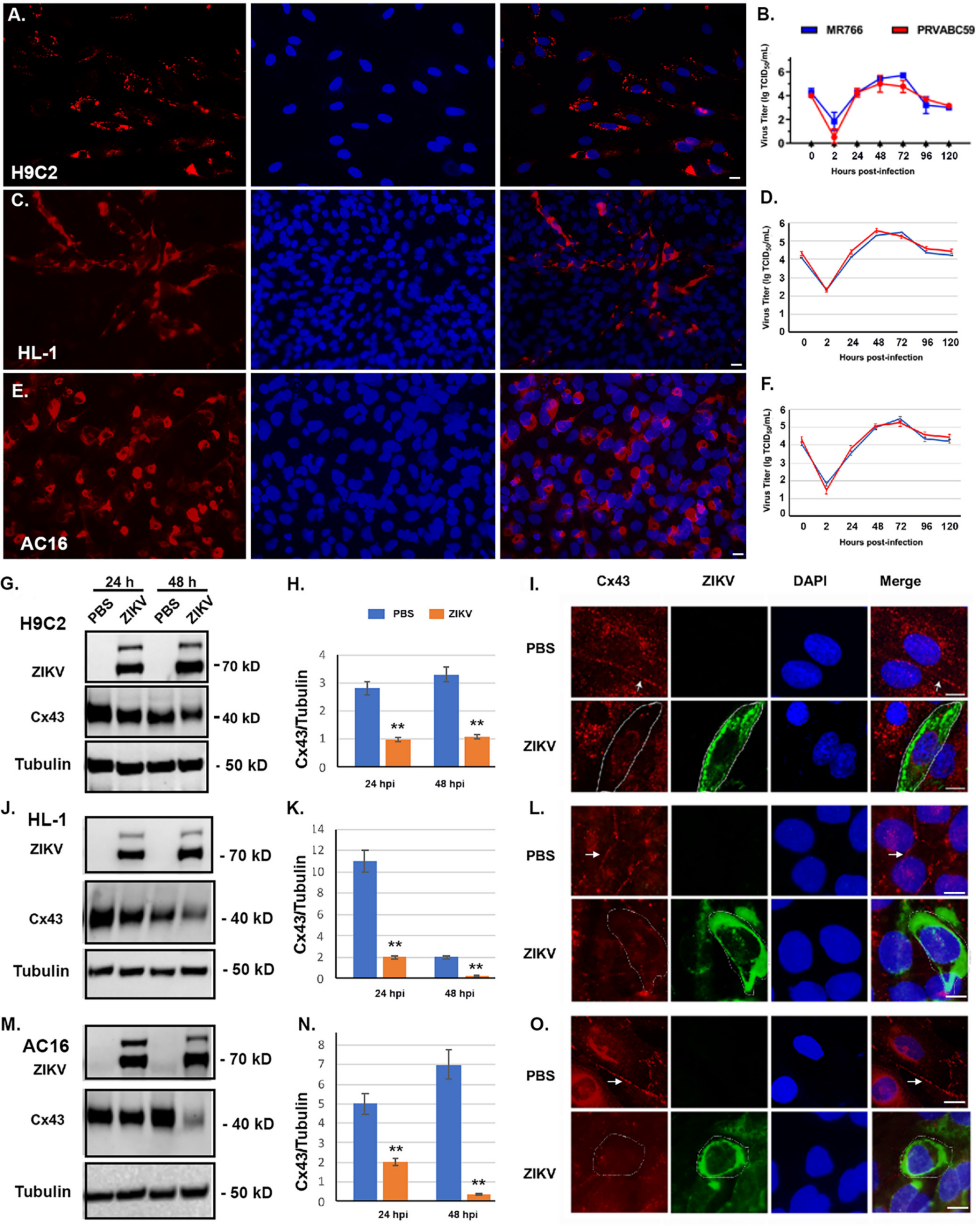
The effects of ZIKV infection on Cx43 in a rat-, mouse-, or human-originated cardiomyocyte. A, C, and E. Infection of ZIKV PRVABC59 at an MOI of 0.5 and IFA to demonstrate the production of ZIKV NS3 protein in H9c2 (A), HL-1 (C), and AC16 (E) as shown in red (left), DAPI to show nuclei (middle), and merged on right. B, D, and F. A viral growth curve was determined for both PRVABC59 and MR766 infection in the H9c2 (B), HL-1 (D), or AC16 (F) cell lines by TCID_50_ assay. G, J and M. Western blot assays were performed to analyze the whole-cell lysate samples at 24 and 48 hpi using anti-Cx43, -NS3, and -tubulin antibodies in H9c2 (G), HL-1 (J), and AC16 (M) cell lines. H, K and N. The Cx43 bands were normalized with the respective tubulin bands and the ratios from three independent Western blot assays were averaged for H9c2 (H), HL-1 (K), or AC16 (N). **, *P* < 0.01. I, L and O. An IFA assay was performed to show the Cx43 between the H9c2 (I), HL-1 (L), or AC16 (O) cells: mock group cells (upper panels) and infected cells (lower panels). Scale bar = 10 μm.

We previously demonstrated that ZIKV can productively infect many types of mammalian cells (25), but it is unknown whether cardiomyocytes are permissive for ZIKV infection. To determine the permissiveness of cardiomyocytes for ZIKV, we infected the rat-, mouse-, or human-originated cardiomyocyte, H9c2, HL-1, or AC-16, respectively, with ZIKV at a multiplicity of infection (MOI) of 0.5. As shown in Fig. 1A, C, and E, immunofluorescent assay (IFA) showed that infection of ZIKV PRVABC59 at an MOI of 0.5 for 24 h resulted in production of the viral protein, NS3, in H9c2, HL-1, or AC-16, respectively. No differences were seen in the infected cells by IFA between MR766 and PRVABC59 strains. Then, 50% tissue culture infectious dose (TCID_50_) assays were performed to determine the viral growth in the three different origin cardiac muscle cells. Three independent TCID_50_ assays were statistically averaged, and viral titers were calculated to be shown as a growth curve. As shown in Fig. 1B (H9c2 cells), Fig. 1D (HL-1 cells) and Fig. 1F (AC16 cells), both MR766 and PRVABC59 strains productively grew in the infected cardiac muscle cells. Therefore, *in vitro* studies demonstrated that cardiomyocytes are permissive for ZIKV infection.

Next, we infected different cardiomyocytes at an MOI of 0.5 and collected whole-cell lysate samples at 24 and 48 h postinfection (hpi) for Western blot assays using anti-Cx43, -NS3 and -tubulin antibodies. The same volume of phosphate-buffered saline (PBS) was used instead of ZIKV PRVABC59 as a mock control. The results were shown in Fig. 1G and H for H9c2 cells, Fig. 1J and K for HL-1 cells, and Fig. 1M and N for AC16 cells. ZIKV infection was shown by the detection of NS3 protein, and Cx43 was significantly reduced by ZIKV infection in both 24 and 48 hpi. Three independent experiments were performed, and one representative blot was shown for each cell line. The Cx43 bands were normalized with the respective tubulin bands and the ratios from three independent Western blot assays were averaged and were shown in Fig. 1H, K, and N, respectively. As can be seen, the Cx43 level was significantly reduced by ZIKV infection.

An IFA assay was then performed to show the Cx43 between the cells. As shown in upper panels of Fig. 1I, L, and O, the Cx43-formed line-like structure was clearly shown (white arrows) in the mock group cells. However, the structure was disrupted by ZIKV infection, as shown in the lower panels of Fig. 1I, L, and O. As outlined in a white line for the infected cell, Cx43 density was also lower than that in its neighboring uninfected cells (Fig. 1I, L, and O, lower panels). These results are consistent with the Western blot results showing that Cx43 protein levels were reduced by ZIKV infection.

Therefore, ZIKV productively infects H9c2, HL-1, and AC16 cell lines. Its infection caused reduction of Cx43 level and disrupted the organization of Cx43 between cardiomyocytes.

### ZIKV infection-caused decrease of Cx43 protein can be rescued by MG132.

**FIG 2:**
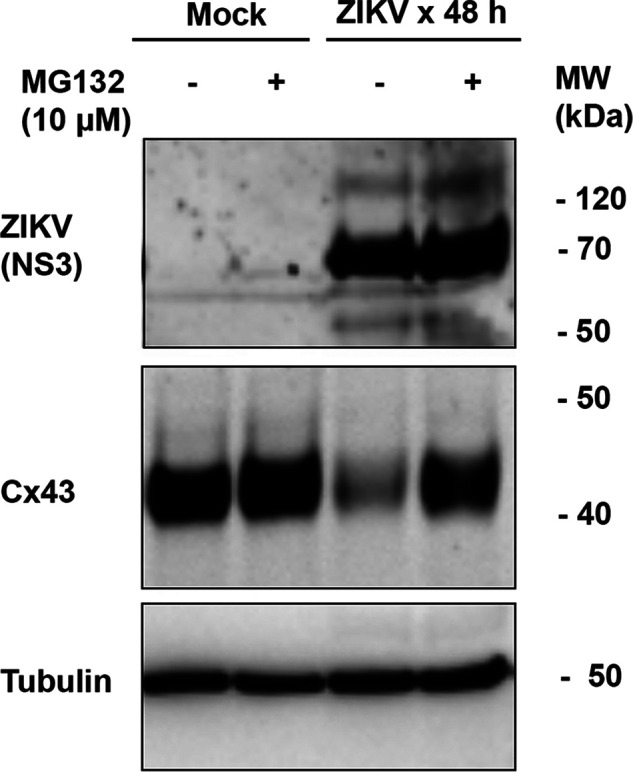
Western blot assay showing the effects of MG132 on ZIKV-induced degradation of Cx43. HL-1 cells were infected with ZIKV PRVABC59 at an MOI of 0.5 for 40 h, then MG132 was added at a working concentration of 10 μM as indicated. The cells were incubated for another 8 h and the whole-cell lysates were collected for Western blot assay using the anti-Cx43, -NS3, and -tubulin antibodies. The image was selected from three independent Western blot assays.

The ubiquitin-mediated protein degradation in ZIKV infected cells was previously reported (26, 27). In search for the mechanism of how ZIKV causes reduction of Cx43 in the cardiomyocytes, we infected mouse cardiomyocytes (HL-1 cells) with ZIKV PRVABC59 at an MOI of 0.5 for 40 h, then MG132, a ubiquitin-proteasome process inhibitor, was added at a working concentration of 10 μM as indicated in Fig. 2. The cells were incubated for another 8 h and the whole-cell lysates were collected for Western blot assay using the anti-Cx43, -NS3, and -tubulin antibodies. As can be seen in Fig. 2 by comparing the Cx43 bands between lanes 1 and 2, MG132 didn’t affect levels of Cx43 in the mock groups. ZIKV infection significantly reduced the level of Cx43, as shown in lane 3. However, as shown in the lane 4, adding MG132 increased the level of Cx43 compared to that of lane 3. Therefore, the ZIKV-induced decrease of Cx43 may be through ubiquitination-mediated proteasomal activities.

### ZIKV infection in 3- to 4-week-old A129 mice causes ZIKV syndrome and is fatal.

**FIG 3:**
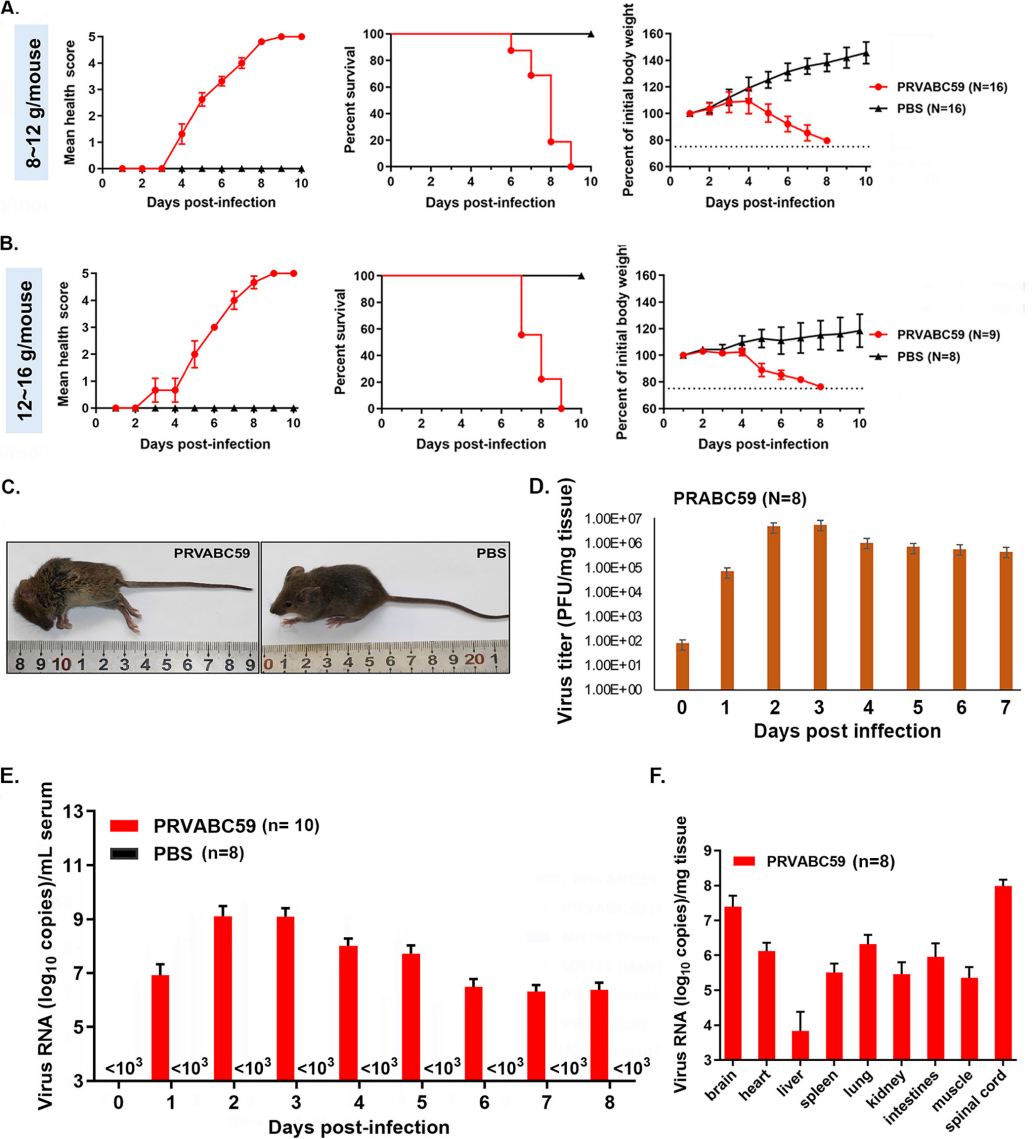
ZIKV PRVABC59 infection in A129 mice. A and B. Mice were weighted to be 8 to 12 g/mouse (*n* = 16) (A) or 12 to 16 g/mouse (*n* = 8) (B) and intraperitoneally injected with 10^5^ TCID_50_ PRVABC59 strain of ZIKV or the same volume of PBS (as control) per mouse. Health scores (left), mouse deaths (middle), and body weights (right) were measured every day. (C) A representative picture to show symptoms of the mice after ZIKV infection, including lethargy and inactivity, wasting, limb weakness or paralysis, shrugging fur, and arched back at 7 dpi. (D) The viral replication in heart tissue was determined by PFU assay, as shown in from 0 to 7 dpi. (E) Dynamic RNA levels of ZIKV PRVABC59 in sera. Total RNA was isolated from mouse blood samples at the indicated days postinfection (dpi) and applied for RT-qPCR (10 mice for each dpi of the ZIKV-infected group or 8 for the PBS-injected group). (F) Eight mice were infected with ZIKV PRVABC59 and euthanized at 8 dpi to isolate organs as indicated. Total RNA was extracted and ZIKV RNA levels in different organs were measured by RT-qPCR.

The WT adult mouse is resistant to ZIKV infection, although it is important for ZIKV study (10–12). The IFN-α/βR^−/−^ (A129) strain mouse, lacking receptors for type I IFN (IFN alpha and beta), is not susceptible to stimulation of IFN-alpha and -beta, and is vulnerable to ZIKV infection (14). To set up the A129 mouse model for ZIKV infection, we intraperitoneally infected the mice (3 to 4 weeks old) with 10^5^ TCID_50_ PRVABC59 strain of ZIKV or the same volume of PBS (as control) per mouse (Fig. 3A and B). The body weights of the mice were measured every day and clinical signs were recorded after infection.

Based on the body weight, two experimental groups of the mice were divided as shown in Fig. 3: 8 to 12 g/mouse (Fig. 3A) and 12 to 16 g/mouse (Fig. 3B). In both groups, ZIKV infection resulted in decreasing of body weights and worsening of the health scores compared to the mice injected with the same volume of PBS. The infected A129 mice all died within 10 days postinfection (dpi). The symptoms of the mice after ZIKV infection include lethargy and inactivity, wasting, limb weakness or paralysis, tremors, shrugging fur, arched back, moribund, and death (Fig. 3C).

Interestingly, no significant differences were detected between the two body weight groups: 8 to 12 g/mouse (Fig. 3A) versus 12 to 16 g/mouse (Fig. 3B), while they were infected with the same number of viruses.

**FIG 4:**
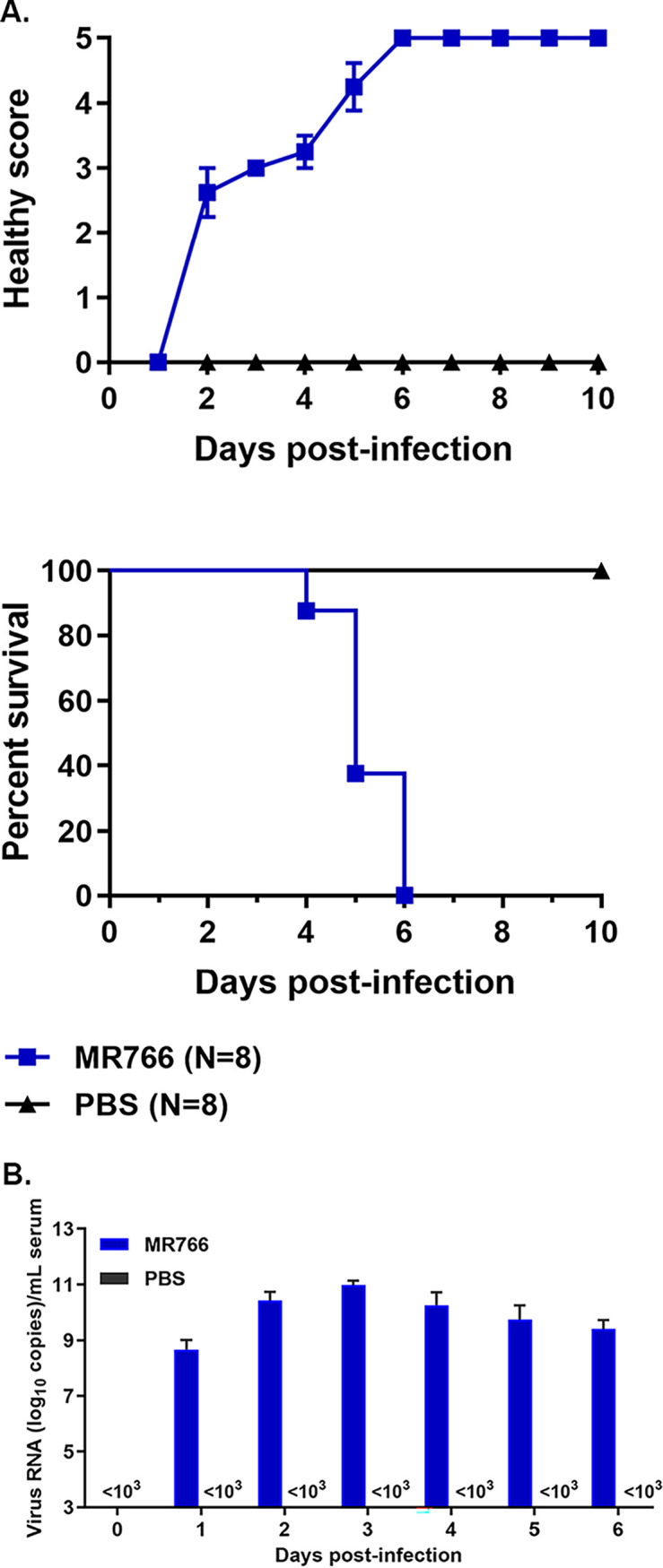
ZIKV MR766 infection in A129 mice. (A) Mice were weighted to be 8 to 12 g/mouse (*n* = 8) and intraperitoneally injected with 10^5^ TCID_50_ MR766 strain of ZIKV or the same volume of PBS (as control) per mouse. Health scores (upper) and mouse deaths (lower) were measured every day. (B) ZIKV MR766 RNA levels in sera. Total RNA was isolated from mouse blood samples at the indicated dpi and applied for RT-qPCR (10 mice for each dpi of either the ZIKV-infected or the PBS-injected group).

PRVABC59 represents the Asian strain that was thought to be more pathogenic to humans than the African strains. Therefore, we also performed the similar experiments using the African strain ZIKV, MR766. However, as can be seen in Fig. 4A, the MR766 strain infection in the A129 mice caused similar consequences to those by PRVABC59. Although previous studies showed that PRVABC59 exhibited more pathogenic effects than MR766 by animal studies or the clinical observations (16, 28), it has been under debate whether the Asian strain is more virulent than African strain because some animal studies observed ZIKV MR766 is even more pathogenic to mice than PRVABC59 (29, 30). This may be caused by using different number of viruses for infection, which needs further detailed investigations.

### ZIKV replicates in multiple organs, including the heart, to generate high viral loads.

Next, we sought to examine whether ZIKV replicates in the A129 mice. Eight mice (3 to 4 weeks old, female and male) were included in each group and they were either infected with PRVABC59 (Fig. 3D to F) or MR766 (Fig. 4B) or injected with the same volume of PBS intraperitoneally. The viral replication in heart tissue was determined by assay as shown in Fig. 3D from 0 to 7 dpi. Our results showed that PRVABC59 ZIKV replicated within 24 hpi and reached a peak at 2 to 3 dpi, then maintained at a high level. All the mice died within 10 dpi. The viral RNA replication in animals was examined by RT-qPCR. First, we isolated the total RNA from the sera of mice at different dpi (from day 0 through day 8). As shown in Fig. 3E and 4B, viral RNA levels in sera increased from 1 dpi and reached a peak at 2 to 3 dpi; in the studies, we set limit of detection of qPCR to 10^3^ copies/mL. Our results suggest that ZIKV can replicate in the infected A129 mice.

Then, we asked whether ZIKV could replicate in different organs of the infected mice. To address this question, we euthanized the PRVABC59-infected mice at 8 dpi and isolated the organs to extract the total RNA for RT-qPCR. As shown in the Fig. 3F, the viral RNA levels were detected in different organs with highest to lowest as shown from spinal cord, brain, lung, heart, intestine, spleen, kidney, muscle, to liver. Since the Asian strain may be more important in the pathogenesis to humans, in the following studies we employed the PRVABC59 strain to complete our animal and *in vitro* studies.

Our results that ZIKV infected A129 mice, replicated in multiple organs of the A129 mice, and caused severe symptoms and deaths are consistent to the previous studies (15, 19, 22, 31). A129 mice in our laboratory support the infection of either Asian or African strain of ZIKV. Therefore, we established an A129 mouse model for studying ZIKV pathogenesis.

### The biomarkers of acute myocardial injury in serum are upregulated by ZIKV infection.

As shown in Fig. 3A, 3B, and 4A, ZIKV-infected A129 mice all die within 10 dpi. We wondered how ZIKV infection causes death of the A129 mouse. Here, we wanted to know whether ZIKV infection in A129 mice could have pathogenic effects on their hearts.

**FIG 5:**
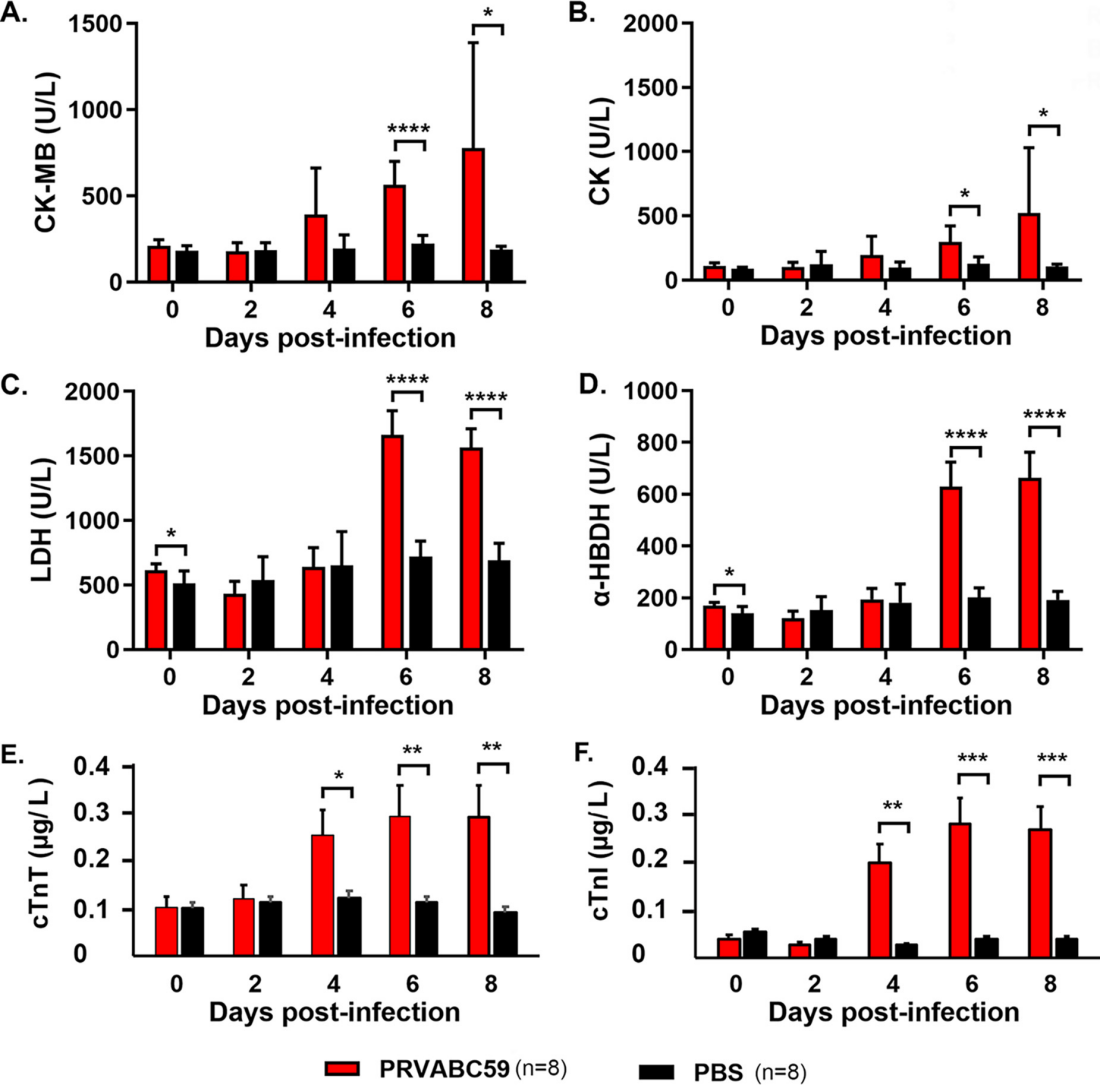
Cardiac muscle enzyme detection assay. (A to D) Levels of CK-MB, CK, LDH, and α-HBDH in the serum collected from mice were measured by a Mindray BS-200 analyzer according to the manufacture’s protocols and expressed in U/L. (E to F) The levels of cTnT and cTnI were measured by electrochemiluminescence assays. Significant (*), *P* < 0.05; very significant (**), *P* < 0.01; highly significant (***), *P* < 0.001; extremely significant (****), *P* < 0.0001.

We examined the serum concentrations of cardiac muscle enzymes that have been defined as the biomarkers of heart diseases such as acute myocardial injury. These enzymes include CK-MB, CK, LDH, and α-HBDH (32–35). PBS-treated mice were used as the control. Both the ZIKV-infected and PBS-treated group contained 8 mice at each time point. CK-MB and CK were significantly increased at 6 dpi in ZIKV-infected mice compared to the PBS-treated mice (Fig. 5A and B). The increased CK-MB and CK reached a peak at 6 and 8 dpi. The other cardiac muscle enzymes, LDH and α-HBDH, also increased at 6 dpi and 8 dpi (Fig. 5C and D).

CK-MB isoform and cardiac troponin are the two commonly used biomarkers for diagnosis of acute myocardial injury. Cardiac troponin appears more accepted by the clinicians so cardiac troponin replaced CK-MB as the biomarker of choice for diagnosing the acute myocardial infarction since 2000 (33, 36). Therefore, we also examined the serum concentrations of cTnT and cTnI. Fig. 5E and F demonstrate that both cTnT and cTnI are significantly elevated from 4 dpi.

### Gap junctions of the intercalated disc are damaged by ZIKV infection.

**FIG 6:**
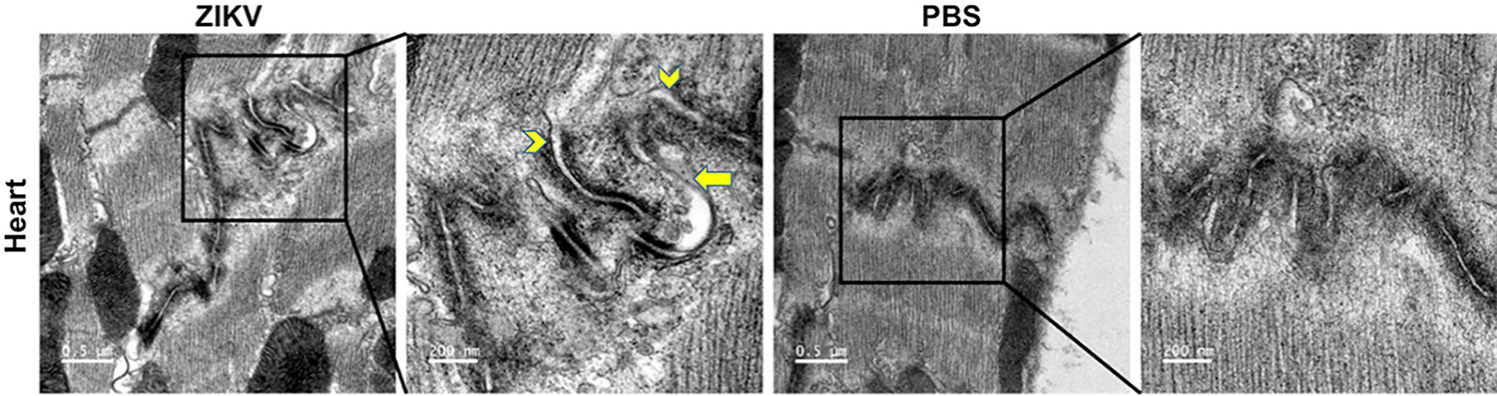
Transmission electronic microscopy to show the intercalated disc (ICD). The ventricle regions from ZIKV-infected (left) or PBS-treated (right) mouse heart tissues were dissected and embedded in Embed 812 kits. The ultrathin sections were stained with aqueous uranyl acetate and lead citrate and observed under a Tecnai G2 Spirit transmission electron microscope. More than 20 images were analyzed, and a representative image is shown. Arrow heads indicate adherens junction; arrow indicates gap junction.

Completing the functional activities of the heart requires the synchronous mechanical and electrical activity of individual cardiomyocytes to ensure the coordinated excitation and contraction. The ICD, a highly organized structure of the myocardium, connects the adjacent individual heart muscle cells to work as a single functional syncytium to fulfill such a function (37). The gap junction is one of the three major complexes of the ICD, connecting the cytoplasm of adjacent cardiomyocytes metabolically and electronically to enable the propagation of electrical stimuli throughout the whole heart. To determine the effect of ZIKV infection on the heart muscle cell organization, we performed electronic microscopy to examine the structure of the ICD. We examined the ICD from 8 ZIKV-infected mice and 8 PBS-treated mice. More than 20 images were analyzed, and a presentative image was selected, as shown in Fig. 6. Although the ICD is folded in a similar pattern in the hearts of both ZIKV-infected (Fig. 6, left) and PBS-injected control mice (Fig. 6, right), the electron density of the ICD plaques is lower in the ZIKV-infected mouse heart than that in control mice. However, the size of the ICD seems expanded, leading to a widened extracellular space at the adherens junction, as shown by the two arrowheads in the ZIKV-infected heart compared to that with narrow extracellular space in the control mice. In addition, some gap junction-like structures with a much thinner and lighter electron density were also observed in the ICD of the ZIKV-infected heart (arrow) compared to the control. Therefore, ZIKV infection altered the structure of the ICD.

### ZIKV infection results in abnormal electrocardiogram (ECG).

**FIG 7:**
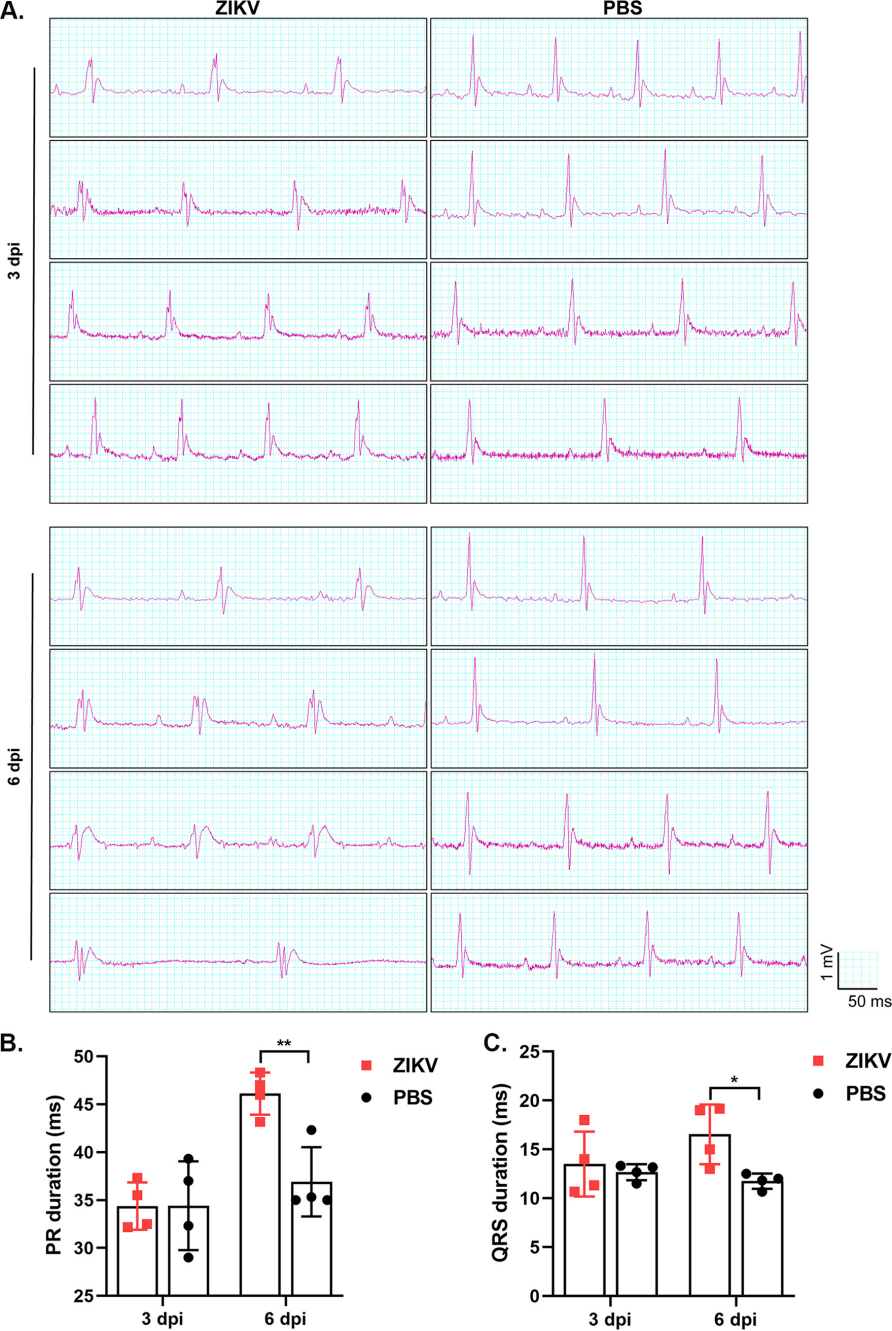
Electrocardiogram (ECG). Mice were weighted to be 8 to 12 g/mouse (*n* = 4) and intraperitoneally injected with 10^5^ TCID_50_ PRVABC59 strain of ZIKV or the same volume of PBS (as control) per mouse. (A) ECG at 3 dpi (upper panel) and 6 dpi (lower panel). (B) Average PR intervals (ms). (C) Average QRS width (ms). *, *P < *0.05; **, *P < *0.01.

Next, we performed functional examinations for the ZIKV-infected mice using ECG. As shown in Fig. 7A, two groups at different time points were set up and 4 mice were included in each group: PBS-treated or ZIKV-infected for 3 days (upper panel) and PBS-treated or ZIKV-infected for 6 days (lower panel). By comparing the ECG of ZIKV-infected mice with that of PBS-treated mice, ECG presented abnormal figurations. At 3 dpi, we can clearly see the fragmented QRS waves, indicating that the ventricular muscle is damaged, resulting in uneven electrical conduction. However, the PR intervals (Fig. 7B) and width of QRS (Fig. 7C) are not significantly affected.

At 6 dpi, besides the fragmented QRS waves, the ECG showed (1) that the PR interval was prolonged (Fig. 7B), which means that the atrial excitation to the ventricular excitation and the conduction time are prolonged, and the conduction is blocked; and (2) that the QRS complex was widened (Fig. 7C) and intraventricular conduction was delayed or blocked. Comparing the ECG presentations of 6 dpi with those of 3 dpi, the ECG showed a larger fragmented QRS complex, wider ventricular QRS complex, and longer PR interval. Furthermore, the heart rate is slower. Therefore, ZIKV infection in A129 mice seriously interfered with their heart function.

### The level of gap junction protein, Cx43, is reduced by ZIKV infection.

The gap junction consists chiefly of three connexins (Cx): Cx43, Cx40 and Cx45 (37). Cx43 has been demonstrated to exist in the cardiac muscle cell of the left and right atrium and the left and right ventricles (38). Therefore, we were curious whether the Cx43 level is affected by ZIKV infection.

**FIG 8:**
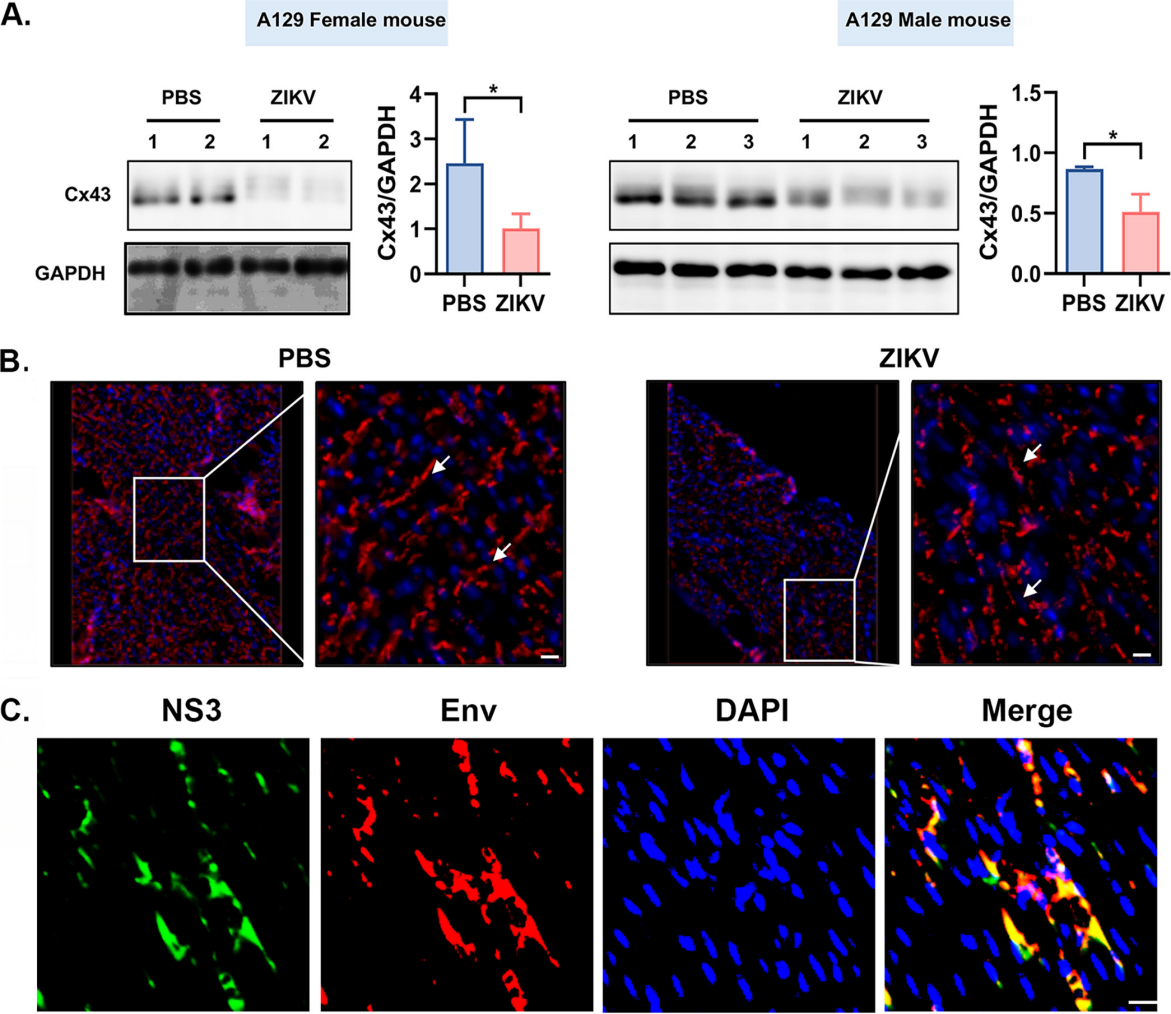
ZIKV infects cardiomyocytes of A129 mice to reduce the level of Cx43 and disrupt Cx43 structures. (A) The ZIKV-infected or the PBS-treated A129 mice were euthanized at 7 dpi and their hearts were isolated and homogenized for Western blot assay to examine the proteins of Cx43 and GAPDH. The average Cx43 levels from ZIKV-infected groups were compared to those from PBS groups. (B) Immunofluorescent assays (IFA) were performed to see if Cx43distribution in the ICD was disrupted in the heart tissue by ZIKV infection. (C) IFA assay to demonstrate the infection of ZIKV in A129 heart tissue. Scale bar = 5 μm.

The ZIKV-infected or the PBS-treated A129 mice were euthanized at 7 dpi and their hearts were isolated and homogenized for Western blot assay, as shown in Fig. 8A, to examine the proteins of Cx43 and GAPDH. Two female and three male mice were used in each group. Obviously, ZIKV infection caused reduction of the Cx43 protein. By comparing the average Cx43 levels from ZIKV-infected groups to those from PBS groups, we revealed that the decrease of Cx43 by ZIKV is statistically significant, as can be seen in the right side of the blot of Fig. 8A.

Immunohistochemistry (IHC) was also performed to see if Cx43 distribution in ICD was disrupted in the heart tissue by ZIKV infection. The PBS-treated cardiac IFA is shown in the left side of Fig. 8B; the Cx43 distributed normally, as shown by the arrows between the cardiomyocytes, but the ZIKV-infected heart tissue showed a less organized and reduced density of Cx43, and the distribution of Cx43 mainly showed a disperse pattern at the ICD (arrows in the right side of Fig. 8B). These results regarding the Cx43 protein in the ZIKV-infected mice were consistent to our experimental findings from the ZIKV-infected neonatal mouse models (data not shown).

Finally, we wanted to know whether ZIKV infects cardiomyocytes *in vivo*. A129 mice were infected with ZIKV, and the heart tissue was frozen and sectioned for immunofluorescent assay (IFA) using anti-NS3 and anti-Envelop antibodies. Figure 8C shows the IFA for the ventricular section of a ZIKV-infected mouse. As can be seen, many ventricular cells were positively stained with anti-NS3 and anti-Envelop antibodies. Therefore, ZIKV infects cardiomyocytes not only in cell culture but also *in vivo*.

## DISCUSSION

Viruses that have been reported to cause cardiovascular abnormalities include Coxsackievirus, parvovirus B19, encephalomyocarditis virus, adenovirus, influenza virus, Epstein-Barr virus, cytomegalovirus, hepatitis C virus, and human immunodeficiency virus (39–42). ZIKV has been recently considered to be another virus being involved in cardiovascular complications, especially in the acute infection phase (43). Bai et al. (44) found that ZIKV can directly infect cardiomyocytes, causing myocarditis and cardiac dysfunction in 4-week-old IFN-α/β receptor knockout mice, but not in wild-type mice. We recently demonstrated that ZIKV infection in neonatal mice not only resulted in abnormal ECG with prolonged PR interval (biomarker of atrioventricular block), widened QRS (biomarker of intraventricular block), and elevated ST wave (biomarker of impairment of myocardium), implying defects of the myocardial functions, but also caused increased levels of CCL2, CXCL9, and CXCL10 that are inducers of cardiovascular diseases and the infarction-like myocardial pathogenesis (data not shown). Furthermore, ZIKV infection caused degradation of the Cx43 protein that is the most abundant isoform of gap junction channels in the ICD; our neonatal mouse model of ZIKV pathogenesis suggests that ZIKV infection causes heart failure, leading to fatal sequelae in ZIKV-caused newborns.

In the present studies, we show that ZIKV causes heart failure in immunocompromised mice (A129). First, 10^5^ TCID_50_ ZIKV caused 100% death of adult A129 mice that exhibited different symptoms, including decreasing body weight, worsening health score, shrugging fur, and paralysis. Moreover, we demonstrated that ZIKV replicated in multiple organs, including spinal cord, brain, heart, intestine, lung, spleen, muscle, kidney, and liver. These results are consistent to those from other groups (15, 19, 22, 31). Then, we were searching for the viral effects on the heart of the A129 mice and found that ZIKV infection resulted in the increase of cardiac muscle enzymes, implicating a viral acute myocardial infarction. Since connexins are required for the heart functional syncytium, we wondered whether Cx43 could be affected by ZIKV infection in adult A129 mice. As we expected, ZIKV infection decreased Cx43 protein (Fig. 1 and 8). Cx43 exists in both atria and ventricles and plays an essential role in the gap junction. Interestingly, our EM assays showed that the ICD in the ventricle between two cardiomyocytes was disrupted (Fig. 6). This disruption is due, at least in part, to the decreased levels of Cx43 protein in the gap junction. It is worth mentioning that ZIKV infection may also decrease the levels of other component proteins in desmosomes and the adherens junction of the ICD, which may mask the changes of the electron density levels in the gap junction and contribute to the overall density decrease of the ICD. Not surprisingly, the Cx43 distribution in the ICD of cardiomyocytes was less organized in the ZIKV-infected mice compared to the PBS-treated mice (Fig. 8), which was also demonstrated in the human-, mouse-, or rat-originated heart cell line (AC16, HL-1, or H9c2, respectively) (Fig. 1). Most importantly, ECG showed that ZIKV infection induced prolonged PR interval, fragmented and widened QRS waves, and slower heart rates (Fig. 7). These ECG figurations occur after myocardial infarction, myocarditis, and other myocardial damage. In summary, we demonstrated that ZIKV infection caused heart failure, which might lead to fatal sequelae in ZIKV-infected adult A129 mice. Combined with the previous studies using the neonatal mouse models, our recent virological and pathogenic investigations of ZIKV using mouse models implicate that ZIKV may be another virus to be involved in viral heart diseases.

Studies stimulated by the 2015 to 2016 pandemic of ZIKV had been focused on the central nervous system (CNS) because ZIKV caused congenital microcephaly in the epidemic areas. *In vitro* and *in vivo* studies have largely stormed into the mechanisms of how ZIKV affects CNS development in the fetus and newborn. The *in vitro* models include 2D- and 3D-cell cultures. The animal models are primarily mouse models and noon-human primates (NPH) models. Primary human Neural progenitor cells (NPCs) (hNPCs), hiNSC-derived NPCs, microglial cell lines, and human dermal fibroblast cells have been used for investigating the effects of ZIKV on cellular function, growth, death, apoptosis, and innate defenses (45). 3D NPCs such as hiPSC-derived cerebral organoid, hiPSC-derived forebrain organoid, and hiPSC-derived cerebral neutrospheres were used to study the viral effects on neural cell differentiation. First, it was found that ZIKV has a wide range of permissive cell types (25) supporting ZIKV’s efficient infection and replication. ZIKV infection causes increased cell death, disrupted cell cycle progression, dysregulated gene expression, attenuated hNPC growth, and apoptosis (46–53). ZIKV replicates in primary human neural progenitors and induces cell death, inhibition of the Akt–mTOR pathway, activation of autophagy, centrosomal depletion, and mitochondrial sequestration of phospho-TBK1 (53, 54). ZIKV preferentially infects NSCs, astrocytes, oligodendrocyte precursor cells, and microglia (55). ZIKV infects NPCs in the ventricle zone (VZ), impairs cell cycle progression, and activates immune responses (56). ZIKV can infect and replicate in primary human trophoblasts (PHTs) (57, 58); PHT cells resist ZIKV infection and release IFN-λ1 (58, 59). Further, increased cell death, reduced proliferation, and decreased neuronal cell layer thickness and overall size were seen in ZIKV-infected 3D culture models (47, 60–63). Lastly, the IFN or the IFN-receptor-deficient mouse models also contributed to the understanding of ZIKV-caused microcephaly.

Other diseases, however, caused by ZIKV have been overlooked. A lot of insights into ZIKV pathogenesis remain to be revealed. ZIKV infection in mice was presented with its replication in multiple organs to have a high production of viral particle and viral RNA in either adult A129 or in WT neonatal mice. This is consistent with the fact that ZIKV productively infects many different types of cell lines (25). Our previous biochemical studies showed that ZIKV infection induces Mib1-mediated degradation of PCM1 that participates in stabilization of centrosomes and microtubule organization (26), and here, we found that ZIKV induced ubiquitination-mediated degradation of Cx43 that is vital for heart syncytia function, modulates rhythmic locomotor activity, and mediates syncytia conduction of the heart (64). IHC studies showed that ZIKV infection in mice induces abnormal distribution of Cx43 in heart tissue. It has been demonstrated that a Cx43 defect is related to heart malformation and sudden infant death syndrome (65, 66). Importantly, Cx43 production is regulated by lamin A and Cx43 stability is maintained by lamin A and tubulin (67, 68). Lamin A abnormality accounts for 10% of dilated cardiomyopathy (68, 69). We found that ZIKV protein interacts with tubulin and lamin A (70). Therefore, we hypothesize that ZIKV infection induces Mib1-mediated proteasomal activity to degrade PCM1 and Cx43 and damage the gap junction, and also impairs lamin A, hence impairing syncytia function of cardiomyocytes.

The information from this study will lead to more studies in the future regarding ZIKV pathogenesis. First, different doses of virus will be used to determine whether the heart failure and deaths are related to infected viral load. Second, genetically attenuated ZIKV will be generated to identify the molecular determinants of viral genome on the pathogenesis of heart failure. This genetically attenuated ZIKV may be applied for safe vaccine development. Lastly, more host proteins will be investigated to see if they are associated with ZIKV-associated heart failure.

## MATERIALS AND METHODS

### Ethics statement.

All animal experiments were conducted strictly in compliance with the animal ethics guidelines and approved protocols. The two experimental protocols were approved by the Animal Care and Use Committee of Xiamen University (acceptance no. XMULAC20160049) and Howard University (protocol no. IACUC-MED-15-01 and animal welfare assurance no. A-3742-01), respectively.

### Cells and viruses.

Vero cells (African green monkey kidney epithelial cells) and H9c2 cells (a myocardial cell line derived from rat heart tissue) were obtained from ATCC and Chinese Academy of Sciences Cell Bank, respectively, and incubated at 37°C with 5% CO_2_ in high-glucose Dulbecco’s modified Eagle medium (DMEM; Gibco, USA) supplemented with 10% fetal bovine serum (FBS; Gibco, USA), 100 μg/mL of streptomycin, 100 U/mL of penicillin, and 2 mM l-glutamine. Human Cardiomyocyte Cell Line AC16 cells were purchased from Sigma-Aldrich Inc. (catalog no. SCC109) and cultured in DMEM/F12 (Sigma, catalog no. D6434) containing 2 mM l-glutamine (EMD Millipore, catalog no. TMS-002-C), 12.5% FBS (EMD Millipore, catalog no. ES-009-B), and 100 μg/mL of streptomycin, 100 U/mL of penicillin. Murine cardiac muscle cell line, HL-1, was purchased from Sigma-Aldrich (catalog no. SCC065) and cultured in Claycomb medium (Sigma) with 10% FBS, 0.1 mM norepinephrine, 2 mM l-glutamine, and 100 μg/mL of streptomycin, 100 U/mL of penicillin.

Two ZIKV strains were used in this study: an Asian lineage ZIKV strain, PRVABC59 (GenBank no. KU501215, isolated in 2015 from a ZIKV-infected patient) (71) and an African lineage ZIKV strain, MR766 (GenBank no. LC002520, isolated in 1947 from a sentinel monkey) (72). Both virus strains were purchased from ATCC, and virus stocks were prepared in Vero cells in our laboratory (18). All virus titers were determined as we previously described (73) and expressed as the 50% tissue culture infectious dose (TCID_50_) based on the Reed-Muench method (74) and stored at −80°C until use.

### Experiment animals.

A129 mice that are deficient in the type I interferon receptor were kindly provided by the Academy of Military Medical Sciences of China or purchased from the Jackson Laboratory (032045-JAX) and bred in a specific-pathogen-free facility. Female and male A129 mice at 3 to 4 weeks of age were used in this study and intraperitoneally (IP) inoculated with ZIKV strain PRVABC59 or MR766 (10^5^ TCID_50_/mouse in a volume of 100 μL). The negative control group was given 100 μL PBS by the same route. Every group contained 8 to10 mice. All mice were monitored at least once per day for clinical signs and mortality, and body weights were recorded once a day. A health score was calculated as follows (18): 0 indicates healthy; 1 indicates lethargy and inactivity; 2 indicates wasting; 3 indicates limb weakness and ruffled fur; 4 indicates neurological symptoms, such as paralysis and tremors; and 5 indicates moribund and death. Mice were undertaken for euthanasia if they lost more than 25% of their weight.

For determination of viremia and virus load in tissues, mice were bled via the retro-orbital sinus under the condition of anesthetization daily. Blood was clarified by centrifugation at 12,000 rpm for 10 min and then the harvested serum was transferred to a sterile tube. Brain, heart, liver, spleen, lung, kidney, intestine, muscle, and spinal cord tissues of PRVABC59-infected A129 mice were aseptically removed and weighted, and then homogenized with a multichannel tissue ball milling apparatus (Scientz-192, Scientz biotechnology, China) in 500 μL PBS and stored at −80°C prior to virus load assays by RT-qPCR or virus titer detection by plaque formation unit (PFU) assay.

### Quantification of ZIKV.

Viral RNA was determined by RT-qPCR, as described previously (18). In brief, total RNA was extracted from the serum and homogenized tissues of ZIKV-infected mice using a GenMagSpin viral DNA/RNA kit (GenMag Bio, China). Then total RNA was reverse-transcribed into cDNA and amplified with a one-step RT-PCR kit (GenMag Bio, China) following the user instructions with primers (forward, TGTCTGACAAAGGCTGGAAA and reverse, AYGACRAAGTCCCACTCTTGAT) and fluorogenic probe (ROX-ATACAGCTCAGCAGRAAGACTTTTGAGA-BHQ2) on a LightCycler 96 real-time PCR system (Roche, Germany). The standard curve of ZIKV RNA copies was determined from 10-fold dilutions of standard sample with known concentrations, and viral RNA copies in the serum and homogenized tissues of mice were calculated.

### Cardiac muscle enzyme detection assay.

Quantitative CK-MB, CK, LDH, and α-HBDH in the serum collected from mice were measured by Mindray BS-200 analyzer according to the manufacture’s protocols (no. 105-000459-00, 105-000458-00, 105-000446-00, and 105-000447-00, respectively; Mindray, China) and expressed in U/L. The levels of cTnT and cTnI were measured by electrochemiluminescence assays, as described by the manufacturer’s protocol (ThermoFisher Scientific, USA).

### Transmission electronic microscopy.

The ventricle regions from mouse heart tissues were dissected, cut into about 3 mm^3^ (1 mm × 1 mm × ~3 mm), and fixed with 2.5% glutaraldehyde in 0.1 M sodium cacodylate buffer immediately at 4°C overnight. Then the samples were postfixed in 1% osmium tetroxide for 1 h and dehydrated in a series of ethanol solutions (50%, 75%, 90%, and 100%), and embedded in Embed 812 kits (Electron Microscopy Sciences, USA). The ultrathin sections were stained with aqueous uranyl acetate and lead citrate and observed under a Tecnai G2 Spirit transmission electron microscope (FEI, USA).

### SDS-PAGE and Western blotting.

Heart tissues of A129 mice were homogenized and lysed, and H9c2, HL-1, and AC16 cells were lysed on ice for 1 h in radioimmunoprecipitation assay (RIPA) lysis buffer (no. P0013B; Beyotime, China) supplemented with 1× protease inhibitors (no. P1005; Beyotime, China). Lysates were clarified by centrifugation at 12,000 rpm for 30 min at 4°C. Supernatants were then collected, mixed with loading buffer (5% 2-mercaptoethanol, 8% glycerol, 2% SDS, 0.01% bromophenol blue, 50 mM Tris pH 6.8), and boiled at 98°C for 10 min. Equal amounts of protein sample were loaded onto 10% SDS-PAGE gels. After separation by electrophoresis in a Mini-PROTEAN Tetra system (Bio-Rad Laboratories, USA), proteins were transferred onto Whatman nitrocellulose membranes with a Trans-Blot Turbo transfer system (Bio-Rad Laboratories, USA). The membranes were incubated in 5% nonfat milk (in PBS) at room temperature for 30 min to block, followed by incubation with primary antibody, including an anti-ZIKV NS3 mouse monoclonal antibody (MAb) 7A9 (produced in our lab), anti-Cx43 antibody (no. C6219; Sigma, USA), and anti-GAPDH antibody (no. 60004-1-Ig; Proteintech, USA) or anti-tubulin antibody (4G1, sc-58666) at 4°C overnight. After three PBST (PBS containing 0.1% Tween) washes, the membrane was incubated with appropriate HRP-conjugated secondary antibodies for 1 h at room temperature. After three washes by PBST, the membrane was incubated with the Pierce ECL Western blotting substrate (no. 34095; ThermoFisher Scientific, USA), and chemiluminescence signals were detected with an ImageQuant LAS4000 scanner (GE Healthcare, USA). The bands were quantified by densitometry using Image J Software (NIH, USA).

### Electrocardiographic (ECG) assessment.

To examine cardiac function of the mice infected with ZIKV or treated with PBS (control), the mice were anesthetized using sodium pentobarbital at 3 or 6 days postinfection (dpi) (*n* = 4/group). Then three electrodes were inserted into the subcutaneous tissue of the mice limbs, and ECG signals of mice were recorded for around 10 min with 1 KHz using a Powerlab workstation (mode no. ML4856; ADInstruments, Castle Hill, Australia) and LabChart 8 software, and 500 ms (ms) of good quality signals were shown in this study.

### Immunohistochemistry (IHC).

Heart tissues from the infected A129 mice and controls were collected at 8 dpi and fixed in 4% paraformaldehyde (PFA) at 4°C overnight, dehydrated with 30% sucrose in PBS, and frozen in optimal cutting temperature (OCT) compound (Leica, UK) for cryosections. The sections of hearts (15 μm thick) were washed three times in PBS, blocked by 2% bovine serum albumin (BSA), and immunostained by anti-Cx43 antibody (no. C6219; Sigma, USA). After rinsing with PBS, the primary antibody was detected by goat antirabbit antibodies conjugated with DyLight 549 (no. 611-142-002; Rockland, USA). Antibodies were diluted in blocking buffer. Finally, nuclei were stained with 4’,6-diamidino-2-phenylindole (DAPI) (no. D1306; Invitrogen, USA) for 5 min. Images of sections were taken under an Axio Imager Z2 microscope (Zeiss, Germany).

### Immunofluorescence assay (IFA).

H9c2, HL-1, or AC16 cells grown in 12-well plates with a circular coverslip inside each well were infected with ZIKV strain of PRVABC59 at an MOI of 0.5. At 24 hpi, the cells were fixed with 4% PFA for 30 min and permeabilized with 0.1% Triton X-100 in PBS for 10 min at room temperature. After incubation in a blocking buffer containing 2% BSA/PBS for 30 min, the cells were stained with a mouse anti-ZIKV NS3 MAb and a rabbit anti-Cx43 protein antibody (no. C6219, Sigma) for 1 h, respectively. Then Alexa Fluor 488-conjugated-goat antimouse antibodies (no. A21202, Invitrogen) and DyLight 549-conjugated goat antirabbit antibodies (no. 611-142-002, Rockland) were used as the secondary antibodies, respectively. Finally, nuclei were stained with DAPI (no. D1306, Invitrogen). The stained cells were observed on an LSM-780 confocal microscope (Zeiss, Germany).

### Viral propagation analyses on H9c2, HL-1, or AC16 cells.

H9c2, HL-1, or AC16 cells were preseeded in 12-well plates and infected with ZIKV strains of PRVABC59 or MR766 at an MOI of 0.1, respectively. At the indicated time points after infection, the cells pellet and cell culture supernatant were collected, freeze-thawing three times, and clarified by centrifugation at 12,000 rpm for 15 min at 4°C. Then the supernatants were collected to detect the virus titers by TCID_50_ assay, as described above.

### Statistical analyses.

Log-rank test was used for the comparisons of survival curves. Two groups were compared by the Student's *t* test. Statistical differences were considered significant for *P* values of <0.05. Significant (*), *P* < 0.05; very significant (**), *P* < 0.01; highly significant (***), *P* < 0.001; extremely significant (****), *P* < 0.0001; ns, not significant. Results were presented as the mean ± standard deviation. “N” refers to the sample size. All statistical analyses were conducted using Prism 8.3.0 software (GraphPad, USA).
